# Pregnancy outcomes of patients with acute fatty liver of pregnancy: a case control study

**DOI:** 10.1186/s12884-020-02980-2

**Published:** 2020-05-11

**Authors:** Lingzhi Chang, Ming Wang, Haixia Liu, Qinghua Meng, Hongwei Yu, Yu-mei Wu, Yunxia Zhu

**Affiliations:** 1 Department of Obstetrics and Gynecology, Beijing Youan Hospital, Capital Medical University, YouAn outer street No.8, Fengtai District, Beijing, 100006 China; 2 Department of Gynecologic Oncology, Beijing Obstetrics and Gynecology Hospital, Capital Medical University, Qihelou street No.17, Dongcheng District, Beijing, 100006 China; 3 Department of Clinical Care Medicine of Liver Diseases, Beijing Youan Hospital, Capital Medical University, Beijing, China

**Keywords:** Acute fatty liver of pregnancy, Intrauterine balloon tamponade, Asphyxia, Postpartum hemorrhage

## Abstract

**Background:**

Limited data exists regarding the pregnancy and infant outcomes of Acute Fatty Liver of Pregnancy (AFLP).

**Methods:**

Retrospectively collected mothers with AFLP and mothers without AFLP in our center from 1/2008 to 6/2018. The primary assessment was to analyze and compare the frequency of negative maternal and fetal outcomes. The secondary assessment was to investigate the role of intrauterine balloon tamponade in reducing negative maternal outcomes.

**Results:**

Compared to 220 matched mothers, 55 AFLP mothers were younger (*P* < 0.001), with fewer pregnancies (*P* = 0.033), complicated with more pregnancy induced hypertension (*P* < 0.001), twins(*P* = 0.002), fetal growth restriction (*P* = 0.044) and male fetus (P < 0.001). 3 (5.5%) of AFLP patients were diagnosed in the postpartum period. Mean gestational week of AFLP diagnosis was 35.25 ± 5.80 weeks. Jaundice (89.1%), nausea or vomiting (58.2%), anorexia (49.1%), fatigue (45.5%) and polydipsia (30.9%) were the main prodromal symptoms. The median duration from diagnosis to delivery was 1.55 ± 4.62 days and 75% (39/52) pregnancy were terminated the pregnancy at the day of diagnosis. 78.8% (41/52) patients received cesarean section, 53.6% (22/41) of which received preventive plasma transfusion before surgery and no one received artificial liver support during the treatment.

In comparison, higher frequency of 16 maternal complications, severe negative outcomes (27.3% vs. 0.9%) and newborn asphyxia (24.6% vs.0.9%) were observed in AFLP population. 3 mothers (mortality rates: 5.5%) died of multiple organ system failure and 6 fetus/infants (death rates: 9.8%) died of distress. When compared to those without negative outcomes, patients with negative fetal outcomes were younger (*P* = 0.042), had more singleton rates (*p* = 0.041), increased mean value of ALT(*P* = 0.011) and T-Bilirubin (*P* = 0.014), decreased prothrombin activity (P = 0.011). Although no statistical significance for the small sample size, there were less refractory postpartum hemorrhage (0% vs.31.3%), hysterectomy (0% vs.12.5%), negative maternal outcomes (16.7% vs.56.3%) in patients underwent intrauterine balloon tamponade when postpartum hemorrhage exceeded 500 ml.

**Conclusions:**

Several symptoms were found to be the main prodromal symptoms of AFLP. Higher frequency of adverse maternal and fetal outcomes was observed in mothers with AFLP than mothers without AFLP. We found five potential risk factors of negative fetal outcomes.

## Background

Acute fatty liver of pregnancy (AFLP) is a rare disease with an incidence of 1 per 7000 to 16,000 pregnancies. It mostly occurs in the third trimester of pregnancy or during early postpartum period [1]. Prompt recognition of the disease and early termination of pregnancy are essential to improve the overall outcome of both mothers and infants. Primipara, male fetus, and multiparous women are considered as risk factors of AFLP [2].Genetic mutation in long-chain 3-hydroxyl coenzyme A dehydrogenase probably leads to abnormal β-oxidation of fatty acids in fetal mitochondria and contributes to microvascular fatty infiltration of the liver of mothers [3]. However, the pathogenesis of AFLP is not yet completely elucidated. Due to better understanding of the disease and the implementation of artificial liver support therapy (ALST) [4] in clinic, as well as the popularization of intensive care unit, the mortality of AFLP for both suffering mothers and infants has dramatically decreased in recent years.

However, AFLP remains a serious disease with high mortality from to 16.5–26.7% in recent study [5] and little data exist regarding the measures to improve outcomes of AFLP. We conducted a comparative study about the negative maternal outcomes (maternal complications and mortality) and fetal/infant outcomes. Also, we evaluated the role of intrauterine balloon tamponade and plasma transfusion by comparing AFLP mothers with and without negative outcomes.

## Methods

### Patient selection and study design

Retrospective analysis of case records was carried out between January 2010 and June 2019 in the departments of Beijing YouAn Hospital, Capital Medical University in China, a tertiary care hospital for liver diseases, including pregnant women diagnosed of AFLP. The relevant institutional ethics review committee approved the trial (approval number: Jing-you-ke-lun-zi[2020]088-hao) and the need for informed consent was waived.

Patients in our clinic and in-patient services were screened and enrolled into AFLP group for the following eligibility criteria: age between 20 and 45 years; clinically diagnosed of AFLP. The diagnosis of AFLP was based on both clinical features and laboratory findings, including: (a) symptoms of anorexia, nausea, vomiting, jaundice, fatigue, cold food preference and abnormal liver function during the third trimester of pregnancy or early postpartum period; (b) characteristic laboratory findings (e.g. elevated alanine transaminase, bilirubin and serum creatinine levels, prolonged prothrombin time, and hypoglycemia); (c) ultrasonography showing fatty liver or liver biopsy sample with characteristic pathological changes; (d) All patients exhibited six or more of the Swansea criteria [6], which objectively confirmed the diagnosis of AFLP. A group of pregnant mothers without AFLP were randomly selected (based on the birth date in the same month and age match) in a 1:4 ratio to serve as the control group. All patients in AFLP group were further assigned to group A if the intrauterine balloon was used to prevent postpartum hemorrhage and group B if not. Key exclusion criteria: evidence of liver failure caused by other reasons including cirrhotic, Wilson disease, activated hepatitis B or C, hemophagocytic lymphohistiocytosis, hepatocellular carcinoma, or cytotoxic drugs.

### Study procedures and data collections

Using an electronic medical record system and paper charts, the following data from the clinic and inpatient services at YouAn Hospital were collected for analysis: baseline information before delivery, including age, gravidity, parity, pregnancy complication; obstetric complications before AFLP, including hypothyroidism during pregnancy, pregnancy induced hypertension (PIH), gestational diabetes (GDM), placenta previa; lab of AFLP onset and the same gestational weeks in non-AFLP patients including platelet, hemoglobin, alanine transaminase (ALT), albumin, total bilirubin, prothrombin activity and creatinine, pertinent physical findings. For women with AFLP, additional data collection was performed that included documentation of gestational weeks at the time ofclinical signs and diagnosis. Data regarding medications, pregnancy complications, and obstetric complications were also collected during or afterdelivery.

### Outcome measurements

Our primary outcomes were the frequency of negative maternal outcomes (maternal complications and mortality) and fetal/infant outcomes. Negative maternal outcomes including obstetrical complications (including placental Abruption, oligohydramnios, meconium stained,postpartum Hemorrhage (500 ml or more),poor wound healing etc), liver failure, renal failure, coagulation disorders, shock and infection. Meconium staining of the amniotic fluid was defined clinically as the presence of meconium at any point during labor and delivery (both thin and thick meconium) by the obstetrical team and documented in the medical record. The aforementioned outcomes will be compared between groups. Our secondary outcome was to identify the predictors of negative outcomes of both mothers and fetus/infants. Also we evaluated the role of intrauterine balloon tamponade in reducing postpartum hemorrhage, hysterectomy and plasma transfusion. The severe negative outcomes of mothers included death, hysterectomy, shock, multiple organ system failure (MOF), hepatorenal syndrome, hepatic encephalopathy stage III-IV, failure to complete recovery, placental abruption grade III-IV, refractory postpartum hemorrhage (2000 ml or more), PTA < 20%, refractory infections (fungi, severe pneumonia, acute pancreatitis). MOF is defined as the failure of 2 or more organs. Negative outcomes of fetal/infant were defined as asphyxia of newborn (Apgar score 0–7) and fetal/infant death (including liver failure, renal failure and coagulation disorders).

### Statistical analysis

Baseline characteristics and laboratory results were summarized for two groups by means of descriptive statistics, including percentage, means ±standard deviation (SD), and 95% CI. For the quantitative variable, the t-test was used to compare group differences. For categorical variables, the chi-square test was used for group comparisons. Significance level was set at *P* < 0.05; all data were analyzed by SPSS 23.0 (SPSS, IBM., New York).

## Results

### Study population and baseline values

**Fig. 1:**
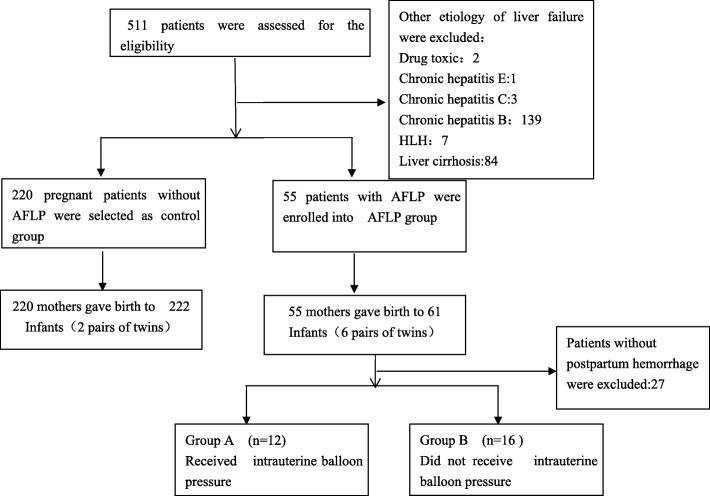
Disposition of Mothers and Infants. **AFLP:**Acute fatty liver of pregnancy**; HLH:** hemophagocytic lymphohistiocytosis

The medical records of 511 pregnant women in our center were reviewed for enrollment. Among them, 55 of 236 patients were diagnosed and assigned to AFLP group. The details satisfied **Swansea criteria** were shown in suppl Table 1.The number of deliveries were about 214,209 from 2009 to 2018 and the ratio of AFLP was 2.56/1000 in our hospital,which was higher than common population.220 patients without AFLP or other liver diseases were selected and assigned to control group. The patients disposition was shown in Fig. 1.

The clinical characteristics of patients in each group was shown in Table 1. When compared to mothers without AFLP (control group), patients with AFLP were significantly younger (28.53 ± 4.71vs.31.31 ± 4.20,*P* < 0.001). Rates of pregnancy induced hypertension (21.8% vs. 2.7%, P < 0.001), twins (10.9%vs.1.4%, *P* = 0.002), fetal growth restriction (FGR) (7.3% vs. 1.4%, *P* = 0.044) and male fetus (96.4% vs 55%, P < 0.001) before the diagnosis of AFLP were higher in AFLP group than control group. The incidence of gestational diabetes seems less in AFLP group than in control group (5.5% vs.13.2%), but was not significant(*P* = 0.11). There were no difference of other pathological pregnancy between groups. The mean value of ALT (219.18 ± 240.11 vs. 14.35 ± 27.65), total bilirubin (160.83 ± 112.54 vs. 9.33 ± 5.44), TBA (89.45 ± 56.89 vs. 3.26 ± 4.11) and CRE (162.91 ± 89.84 vs. 46.83 ± 11.62) were significantly higher in AFLP group than in control. And the mean value of PLT (130.53 ± 70.16 vs. 205.35 ± 56.03), HGB (104.27 ± 23.44 vs. 120.13 ± 12.40), albumin (25.54 ± 4.67 vs. 33.26 ± 3.99) and prothrombin activity (40.44 ± 23.37 vs. 119.05 ± 13.23) were significantly lower in AFLP group than in control. Also, the incidence of hypoglycemia (61.8% vs.20.9%) was significantly higher in AFLP group than in control.

**Table 1:** Baseline characteristics of mothers with and without AFLP(*n* = 275)

	AFLP Group (*n* = 55)	Control Group (*n* = 220)	t/χ2/Z, P
**Age (mean ± SD, years)**	28.53 ± 4.71	31.31 ± 4.20	t = 4.3, *P* = 2.8*10^−6^
**Gravidity, n (%)**			
1	28 (50.9)	89 (40.9)	Z = 1.7, *P* = 0.087
2	16 (29.1)	62 (28.2)	
> 2	11 (20)	69 (30.1)	
**Multiparae, n (%)**	22 (40)	87 (39.5)	**χ2** = 0.004, *P* = 0.951
**Complications before the AFLP onset, n (%)**			
Hypothyroidism during pregnancy	2 (3.6)	9 (4.1)	*χ2 = 0, *P* = 1
PIH	12 (21.8)	6 (2.7)	χ2 = 26.2, *P* = 3.1*10^−7^
GDM	3 (5.5)	29 (13.2)	χ2 = 2.5, P = 0.11
Placenta previa	2 (3.6)	4 (1.8)	*χ2 = 0.096, *P* = 0.76
Twins	6 (10.9)	3 (1.4)	*χ2 = 9.8, P = 0.002
FGR	4 (7.3)	3 (1.4)	*χ2 = 4.0, P = 0.044
Fetal gender (male)	53 (96.4)	121 (55)	*χ2 = 32.3, *P* = 1.3*10^− 8^
**Lab on first visit (mean ± SD)**			
Platelet (*10^9^/L)	130.53 ± 70.16	205.35 ± 56.03	t = 8.4, *P* = 4.4*10^−15^
Hemoglobin(g/L)	104.27 ± 23.44	120.13 ± 12.40	t = 4.8, *P* = 9.0*10^−6^
ALT (IU/L)	219.18 ± 240.11	14.35 ± 27.65	t = 6.3, *P* = 5.2*10^−8^
Serum Glucose (umol/L)	4.00 ± 1.57	4.67 ± 2.82	t = 1.4, *P* = 1.2
Hypoglycemia (%)	34 (61.8)	46 (20.9)	*χ2 = 35.7, *P* = 2.3*10^−9^
Hyperglycemia(%)	4 (7.3)	8 (3.6)	*χ2 = 0.66, *P* = 0.42
TBA (umol/L)	89.45 ± 56.89	3.26 ± 4.11	t = 9.7, *P* = 4.6*10^−12^
Albumin(g/L)	25.54 ± 4.67	33.26 ± 3.99	t = 12.1, *P* = 8.9*10^−27^
Total Bilirubin (umol/L)	160.83 ± 112.54	9.33 ± 5.44	t = 10.0, *P* = 7.2*10^−14^
Prothrombin activity(%)	40.44 ± 23.37	119.05 ± 13.23	t = 23.8, *P* = 7.3*10^−33^
Creatinine (umol/L)	162.91 ± 89.84	46.83 ± 11.62	t = 9.6, *P* = 3.0*10^−13^

*Continuety correction;** Fisher’s exact test; AFLP:Acute Fatty Liver of Pregnancy; ALT, alanine aminotransferase; TBA, total bile acid

### Characteristics and outcomes of the AFLP patients in our center

The clinical characteristics of the AFLP patients from the chosen studies were summarized in Supplemental Table 1. Among all patients of AFLP group, 3 (5.5%) were diagnosed in the postpartum period. The mean gestational week was 35.25 ± 5.80 weeks in patients diagnosed before delivery and the mean days were 2.33 ± 0.57 days in patients diagnosed after delivery. The median duration from diagnosis to delivery was 1.55 ± 4.62 days and 75% (39/52) pregnancies were terminated at the day of diagnosis. More patients received cesarean section (74.5% vs.49.5%) to terminate the pregnancy. 53.6% (22/41) of AFLP patients received preventive plasma transfusion before surgery. Mean gestational weeks at delivery were 36.26 ± 2.58 weeks in AFLP group, which were significant earlier than control(*P* = 5.47*10^− 10^). During perinatal period, more patients intrauterine balloon tamponade (21.8% vs.3.2%, *P* < 0.05), preventive plasma transfusion (40% vs.0%, P < 0.05), hysterectomy (or uterine artery embolism) (5.5% vs.0%, P < 0.05), blood transfusion (61.8% vs.1.8%, P < 0.05) and intensive care unit admission (61.8% vs.0.9%, P < 0.05) in AFLP group than control. No one received artificial liver support or liver transplantation during the treatment.

As is show in Table 2, there were a significantly higher frequency of obstetrical complications in AFLP group than control: placental abruption (12.7% vs.0.5%), meconium stained (II-III)(40% vs.8.6%), and postpartum hemorrhage (52.7% vs.12.3%). No difference was found of oligohydramnios (7.3% vs.6.4%) between two groups. After termination of pregnancy, more patients in AFLP group had problem healing wound of episiotomy or abdominal section than control (14.5% vs. 0.5%, *P* < 0.05). In terms of non-obstetrical complications, 83.6% coagulation disorders, 47.3% acute hepatic failure, 85.5% renal insufficiency, 98.2% rising of total bile acids, 47.3% ascites, 18.2% encephalopathy, 3.6% hepatorenal syndrome,7.3% MOF, 1.8% shock and 12.7% infections (2 fungi infection,1 severe pneumonia, 1acute pancreatitis, 1 bacterial peritonitis,1 biliary tract infection and 1 pressure sore) were found in AFLP group. However, only one patient with slight coagulation disorders and 2.3% patients with rising of total bile acids were found in control. Finally, three mothers in AFLP group were died of multiple organ system failure and 1mother did not completely recovered for two weeks. In terms of fetal/infant complications, a significantly higher frequency of preterm delivery (47.5% vs.5.4%, *P* < 0.05), fetal distress (45.9% vs.3.1%, P < 0.05), asphyxia of newborn (24.6% vs.0.9%, P < 0.05), NICU admission (19.7% vs.1.3%, P < 0.05) and fetal/infant death (9.8% vs.0, P < 0.05) were found in AFLP group than in control group.

**Table 2:** Negative maternal and fetal/infant outcomes in mothers with and without AFLP(n = 275)

	AFLP Group	Control Group	t/χ2, ***P***-value
**Gestational weeks of delivery(mean ± SD, weeks)**	36.26 ± 2.58	39.05 ± 1.49	t = 7.4, *P* = 5.5*10^−10^
**Maternal Complications, n (%)**	**(n = 55)**	**(n = 220)**	
Cesarean section	41 (74.5)	109 (49.5)	χ2 = 11.1, *P* = 0.001
Placental Abruption	7 (12.7)	1 (0.5)	χ2 = 23.5, *P* = 1.0*10^−6^
Oligohydramnios	4 (7.3)	14 (6.4)	χ2 = 0.059, *P* = 0.81
Meconium stained	22 (40)	19 (8.6)	χ2 = 34.1, P = 5.2*10^−9^
Postpartum Hemorrhage	29 (52.7)	29 (12.3)	χ2 = 44.4, *P* = 2.7*10^−11^
Intrauterine balloon pressure	12 (21.8)	7 (3.2)	χ2 = 23.8, P = 1.0*10^−6^
Hysterectomy or UAE	3 (5.5)	0 (0)	**P = 0.008
Need Blood Transfusion	34 (61.8)	4 (1.8)	χ2 = 133.0, P = 9.0*10^−31^
Poor wound healing	8 (14.5)	1 (0.5)	χ2 = 23.3, P = 1.0*10^− 6^
ICU admission	34 (61.8)	2 (0.9)	χ2 = 143.5, P = 4.6*10^−33^
Coagulation disorders	46 (83.6)	1 (0.5)	χ2 = 214.9, P = 1.2*10^−48^
Acute hepatic failure	26 (47.3)	0 (0)	χ2 = 114.9, *P* = 8.5*10^−27^
Renal insufficiency	47 (85.5)	1 (0.5)	χ2 = 220.6, *P* = 6.6*10^−50^
Rising of TBA	54 (98.2)	5 (2.3)	χ2 = 240.2, *P* = 3.6*10^−54^
Ascites	26 (47.3)	0 (0)	χ2 = 114.9, P = 8.5*10^−27^
Encephalopathy	10 (18.2)	0 (0)	χ2 = 36.5, *P* = 1.5*10^−9^
Hepatorenal syndrome	2 (3.6)	0 (0)	**P = 0.039
MOF	4 (7.3)	0 (0)	**P = 0.001
Shock	1 (1.8)	0 (0)	**P = 0.20
Infection	7 (12.7)	0 (0)	χ2 = 28.7, *P* = 8.3*10^−8^
Death or insufficency	4 (7.3)	0 (0)	**P = 0.001
**Fetal/newborn**, n (%)	**(*n*** **= 61)**	**(*n*** **= 223)**	**t/χ2, P-value**
Preterm delivery	29 (47.5)	12 (5.4)	χ2 = 68.9, P = 1.0*10^−16^
Fetal distress	28 (45.9)	7 (3.1)	χ2 = 81.1, *P* = 2.2*10^−19^
Asphyxia of newborn (Apgar score < 7at 1 min)	17 (24.6)	2 (0.9)	χ2 = 43.7, *P* = 3.9*10^−11^
Neonatal ICU admission	12 (19.7)	3 (1.3)	χ2 = 28.6, P = 8.9*10^−8^
Fetal death	6 (9.8)	0 (0)	χ2 = 17.9, P = 2.3*10^−6^

*Continuety correction;** Fisher’s exact test; AFLP,Acute Fatty Liver of Pregnancy; UAE,uterine artery embolism; MOF,multiple orgain failure; TBA, total bile acid. ICU, intensive care unit

When compared to those without negative outcomes, predictors of negative fetus and infants outcomes were younger mothers (27.00 ± 2.57 vs.29.21 ± 5.28), more singleton rates (100% vs.72.73%), higher mean values of ALT (328.80 ± 277.48 vs. 164.60 ± 194.25) and T-Bilirubin (208.46 ± 108.89 vs. 130.63 ± 107.46), lower mean value of prothrombin activity (29.51 ± 23.10 vs. 46.50 ± 22.31) (suppl Table 2). Besides, more patients in this group received preventive plasma transfusion (70.6% vs.27.3%) and intrauterine balloon tamponade (47.1% vs.15.9%). There were no predictors of negative maternal outcomes found in baseline values (suppl Table 3).

### The role of intrauterine balloon tamponade in preventing future postpartum hemorrhage

To evaluate the role of intrauterine balloon tamponade, we stratified 28 patients with postpartum hemorrhage more than 500 ml into **group A** (patients received intrauterine balloon tamponade) and group B (patients received other methods instead of intrauterine balloon tamponade). Compared to patients in group B, less patients were found in group A suffered from refractory postpartum hemorrhage (> 2000 ml in delivery) (0% vs.31.3%, *p* = 0.101), hysterectomy (0% vs.12.5%, *p* = 0.596), negative maternal outcomes (16.7% vs.56.3%, *p* = 0.083). But none of them had statistical significance due to restricted sample size (Table 3).

**Table 3:** Maternal outcomes in patients received intrauterine balloon pressure or not when postpartum hemorrhage exceeding 500 ml(n = 28)

	Goup A (***n*** = 12)	Group B (***n*** = 16)	t/χ^**2**^, P-value
**Age (mean ± SD, years)**	29.75 ± 4.45	29.00 ± 4.58	t = 0.43, *P* = 0.67
**Multiparae, n(%)**	7 (58.3)	5 (31.3)	χ^2^ = 2.1, *P* = 0.15
**PIH, n(%)**	5 (41.7)	3 (18.8)	*χ^2^ = 0.82, *P* = 0.37
**Twins, n(%)**	1 (8.3)	1 (6.3)	*χ^2^ = 0, P = 1
**Lab on first visit (mean ± SD)**			
Platelet (*10^9^/L)	9142.50 ± 68.63	111.51 ± 56.91	t = 1.3, P = 0.20
Hemoglobin(g/L)	118.00 ± 21.23	101.69 ± 20.79	t = 2.0, *P* = 0.052
ALT (IU/L)	305.56 ± 308.20	238.76 ± 272.40	t = 0.61, *P* = 0.55
TBA (umol/L)	3.72 ± 0.75	3.83 ± 1.26	t = 0.24, *P* = 0.82
Hypoglycemia (umol/L)	98.76 ± 66.48	94.60 ± 70.28	t = 0.14, *P* = 0.89
Albumin(g/L)	26.59 ± 4.13	25.80 ± 5.73	t = 0.40, *P* = 0.69
Total Bilirubin (umol/L)	142.68 ± 99.70	216.96 ± 138.24	t = 1.6, *P* = 0.13
Prothrombin activity(%)	48.82 ± 12.77	31.62 ± 26.42	t = 2.3, P = 0.033
Creatinine (umol/L)	134.88 ± 47.93	178.28 ± 97.33	t = 1.4, *P* = 0.17
Cesarean section(%)	12 (100)	11 (68.8)	*χ^2^ = 2.7, P = 0.10
**Outcomes, n(%)**			
Refractory Postpartum Hemorrhage	0 (0)	5 (31.3)	*χ^2^ = 2.7, P = 0.10
Uterine artery embolism	1 (8.3)	0 (0)	*χ^2^ = 0.022, *P* = 0.88
Hysterectomy	0 (0)	2 (12.5)	*χ^2^ = 0.28, *P* = 0.60
Poor wound healing of perineotomy or abdominal section	2 (16.7)	0 (0)	*χ^2^ = 0.91, *P* = 0.34
Negative marternal outcomes	2 (16.7)	9 (56.3)	*χ^2^ = 3.0, P = 0.083
Death of mothers	0 (0)	1 (6.3)	*χ^2^ = 0, P = 1.0

*Continuety correction;** Fisher’s exact test; AFLP:Acute Fatty Liver of Pregnancy; PIH:pregnancy induced hypertension; FGR:fetal growth restriction; ALT, alanine aminotransferase; TBA, total bile acid

## Discussion

AFLP remains a serious disease with high mortality from to 16.5–26.7% [4, 5] due to severe complications such as DIC, renal function impairment, hepatic encephalopathy, hypoglycemia, MOF, etc. [7] Because the scarcity of AFLP [8], more comparative studies are needed about its clinical characteristics, treatment and outcomes.

Due to its characters of quick onset, rapid progression and fetal relation, prompt recognition and early termination of pregnancy are essential to improve the overall outcome of both mothers and infants. Primipara, male fetus, and multiparous women are considered as risk factors of AFLP [2]. Except for these factors, we also found PIH^8^ and FGR were the risk factors of AFLP in Chinese population. AFLP patients seems to had less gestational diabetes (5.5% vs.13.2%) but there was no statistical significance. There is disagreement in the published literature regarding the symptoms and clinical common manifestations of AFLP [9, 10]. We found that jaundice, nausea or vomiting, anorexia, fatigue and cold food preference were the main prodromal symptoms. Fatigue and cold food preference are easily ignored symptoms. Cold food preference is a much more obvious manifestation in China because usually people prefer to have warm food and drink. Previous studies [9–11] found that classical features of ascites or bright liver were only seen in a quarter of patients who underwent abdominal ultrasonography. However, in our patients underwent cesarean section, the rates of ascites were 46.3%. When compared to common patients, the abnormal values of laboratory test such as ALT, CRE, TBA, total bilirubin and PTA might be more severe in patients with AFLP. Although liver biopsy is the gold standard for diagnosis of AFLP, it was not performed in our study and most previous studies due to the invasive nature of this procedure, combined with severe coagulation disorders of patients. Besides, there are sufficient evidence of typical clinical findings fordiagnosis [12, 13].

Except for early diagnosis, immediate delivery and comprehensive supportive treatment remains the mainstay in the management of AFLP. Similar to earlier studies, the common non- obstetrical complications observed among our patients were coagulation disorders (83.6%), acute hepatic failure (47.3%), renal insufficiency (85.5%), rising of total bile acids (98.2%), ascites (47.3%), encephalopathy (18.2%), 12.7% infections (including 2 fungi infection,1 severe pneumonia, 1 acute pancreatitis, 1 bacterial peritonitis, 1 biliary tract infection and 1 pressure sore), 7.3% multiple organ failure and 1.8% shock. In previous study, acute liver failure and acute renal failure are the most significant and life-threatening complications of AFLP [14]. Renal replacement therapy could prevent and reverse the further worsening of acute kidney injury. A rtificial liver support therapy has been widely used in the management of acute or chronic liver failure caused by various etiologies, which, to some extent, alleviates liver injury and provides a homeostatic environment for hepatocyte regeneration [15, 16]. In our center, no patients received ALST, however, 40% percent patients received plasma Transfusion at the time of their admission. Timely termination of pregnancy and plasma transfusion could improve clinical outcomes of most patients. Artificial liver support therapy is a quick onset and rapid progression disease. Acute liver failure in several hours to several days affect synthetic function, including coagulation factors and fibrinogens. In previous study, potential factors influencing adverse maternal outcomes were male fetus, postpartum diagnosis of AFLP, intrauterine fetal death, disseminated intravascular coagulation, prothrombin time and activated partial thromboplastin time. The factors could be improved were coagulation disorders, including disseminated intravascular coagulation, prothrombin time and activated partial thromboplastin time. Intermittent 800-1200 ml plasma transfusion could supply coagulation factors, and guaranteed a safe surgery when immediate termination was needed. After removing risk factors of AFLP, the disabled liver cells recovered quickly. The mortality rates were 5.6–26.7%% in previous studies in patients received ALST [4, 5]. The 5.5% mortality rates in our study were not only lower previous study with artificial liver support therapy, but also significantly lower than the 85% in 1980 before the advent of this technique [16], suggesting that timely preventive plasma transfusion and immediate surgery is an effective method in improving survival of AFLP patients. All 3 deaths in our center caused by multiple organ failure occurred in 2008–2010. At that time comprehensive support was immature. Furthermore, all survived patients were administered from 2010 to 2019, which indirectly suggesting the importance of comprehensive support in the management of AFLP.

Another study revealed 2 complications, postpartum hemorrhage and multiple organ dysfunctions, were associated with the outcomes of AFLP patients during postpartum. Our study evaluated the role of intrauterine balloon tamponade, we stratified the 28 patients with postpartum hemorrhage exceeding 500 ml. Compared to patients who received other methods to control postpartum hemorrhage, intrauterine balloon tamponade could prevent refractory postpartum hemorrhage (> 2000 ml)(0% vs.31.3%), hysterectomy (0% vs.12.5%), negative maternal outcomes (16.7% vs.56.3%). However, due to the limited sample size, there were no statistical significances in the above complications.

Given the retrospective nature of our study, we were unable to determine the long term outcomes of AFLP. Besides, details between the risk factors and negative fetal/infant outcomes could not be explained. Also, since its low incidence, multicenter comparative studies were needed to verify the value of preventive plasma transfusion and postpartum intrauterine balloon tamponade in improving the outcomes of mothers.

## Conclusion

In conclusions, the main prodromal symptoms of AFLP were jaundice, nausea or vomiting, anorexia, fatigue and cold food preference among our patients. Higher frequency of adverse maternal and fetal/infant outcomes was observed in mothers with AFLP when compared to mothers without AFLP. We found that younger mother, singleton pregnancy, higher mean values of ALT and T-Bilirubin, lower mean value of prothrombin activity were the potential risk factors for negative fetal/infant outcomes. Intrauterine balloon tamponade may improve maternal outcomes, but need further verification.

## Supplementary information

**Additional file 1: Suppl Table 1.** The rates of Swansea criteria for the diagnosis of AFLP (*n* = 55).

**Additional file 2:Suppl Table 2.** Predictors of Negative mother Outcomes in mothers with AFLP(n = 55)

**Additional file 3 Suppl Table 3.** Maternal outcomes in patients received intrauterine balloon pressure or not when postpartum hemorrhage exceeding 500 ml(*n* = 28)

## Abbreviations

AFLP: Acute fatty liver of pregnancy
ALT: Alanine aminotransferase
ALST: Artifificial liver support therapy
PIH: Pregnancy induced hypertension
GDM: Gestational diabetes
UAE: Uterine artery embolism
MOF: Multiple orgain failure
TBA: Total bile acid
ICU: Intensive care unit
