# Supplementary material for: Pregnancy outcomes of patients with acute fatty liver of pregnancy: a case control study
Source: BMC Pregnancy Childbirth. 2020 May 11;20:282. doi: 10.1186/s12884-020-02980-2 (PMC7216501; doi:10.1186/s12884-020-02980-2)
Supplement: Supplementary file 1 — Additional file 1: Suppl Table 1. The rates of Swansea criteria for the diagnosis of AFLP (n = 55). [file 12884_2020_2980_MOESM1_ESM.doc]

**Suppl Table 1. The rates of Swansea criteria for the diagnosis of AFLP (n=55).**

| **Gestational weeks of diagnosis** | **n(%)** |
| --- | --- |
| **Symptoms of diagnosis** |  |
| Jaundice | 49 (89.1) |
| nausea or vomitting | 32 (58.2) |
| Anorexia | 27 (49.1) |
| Fatigue | 25 (45.5) |
| Polydipsia | 17 (30.9) |
| Encephalopathy | 16(29.1) |
| Abdominal pain | 11（20） |
| **Characteristic laboratory findings** |  |
| elevated alanine transaminase (＞ 42 U/L) | 54（98.2） |
| prolonged prothrombin time(PT ＞ 14) | 52（94.5） |
| elevated bilirubin(＞ 17 μmol/L) | 51（92.7） |
| leucocytosis(＞ 11 × 10^9^/L) | 44（80.0） |
| hypoglycaemia(＜ 4 mmol/L) | 34（61.8） |
| elevated serum creatinine levels(Cr ＞ 150 μmol/L) | 28（50.9） |
| Ultrasonography images showing fatty liver | 36 (65.5) |

**AFLP：Acute Fatty Liver of Pregnancy.**
