# Supplementary material for: Pregnancy outcomes of patients with acute fatty liver of pregnancy: a case control study
Source: BMC Pregnancy Childbirth. 2020 May 11;20:282. doi: 10.1186/s12884-020-02980-2 (PMC7216501; doi:10.1186/s12884-020-02980-2)
Supplement: Supplementary file 2 — Additional file 2:Suppl Table 2. Predictors of Negative mother Outcomes in mothers with AFLP(n = 55) [file 12884_2020_2980_MOESM2_ESM.docx]

**Suppl Table 2. Predictors of Negative mother Outcomes in mothers with AFLP(n=55)**

| **Maternal Baseline Characteristics** | **Mothers with Negative Outcomes**  **(n=15)** | **Mothers without Negative outcomes**  **(n=40)** | **t/χ2/Z, p-Value** |
| --- | --- | --- | --- |
| **Age (mean±SD, years)** | 28.67± 5.38 | 28.48± 4.50 | t=0.13, p=0.89 |
| **Gravidity , n(%)** |  |  |  |
| 1 | 9(60) | 20(50) | Z=0.23, p=0.82 |
| 2 | 2(13.3) | 13(32.5) |  |
| >2 | 4(26.7) | 7(17.5) |  |
| **Multiparae, n(%)** | 4(26.7) | 18(45) | χ^2^=1.5, p=0.22 |
| **Gestational weeks of onset symptoms(mean±SD)** | 34.99±2.97 | 36.43±2.32 | t=1.8, p=0.075 |
| **Complications before the AFLP onset, n(%)** | | | |
| PIH | 4(26.7) | 8(20) | χ^2^=0.028, p=0.87 |
| twins | 1(6.7) | 5(12.5) | χ^2^=0.018, p=0.90 |
| FGR | 1(6.7) | 3(7.5) | *χ^2^=0, p=1 |
| **Lab on first visit(mean±SD)** | | | |
| Platelet (*109/L) | 128.80±61.44 | 131.18±73.89 | t=0.11, p=0.91 |
| Hemoglobin(g/L) | 105.73±29.52 | 103.73±21.14 | t=0.28, p=0.78 |
| ALT(IU/L) | 250.13±323.42 | 207.57±204.38 | t=0.58, p=0.56 |
| TBA(umol/L) | 123.48±93.96 | 81.20±41.90 | t=1.2, p=0.25 |
| Hypoglycemia(umol/L) | 4.64±1.79 | 3.79±1.46 | t=1.5, p=0.14 |
| Albumin(g/L) | 25.75±5.78 | 25.46±4.30 | t=0.20, p=0.84 |
| Total Bilirubin(umol/L) | 196.20±154.36 | 147.57±91.22 | t=1.1, p=0.27 |
| Prothrombin activity(%) | 35.08±23.06 | 42.50±23.45 | t=1.0, p=0.30 |
| C[reatinine](http://www.youdao.com/w/creatinine/#keyfrom=E2Ctranslation)(umol/L) | 171.46±99.30 | 159.71±87.17 | t=0.43, p=0.67 |
| **[Cesarean s](http://dict.youdao.com/w/cesarean delivery/" \l "keyfrom=E2Ctranslation)ection, n(%)** | 12(80) | 29(72.5) | χ^2^=0.32, p=0.57 |
| **ICU admission, n(%)** | 11(73.3) | 22(55) | χ^2^=1.5, p=0.22 |
| **Preventive plasma transfusion, n(%)** | 7(46.7) | 15(37.5) | χ^2^=0.38, p=0.54 |
| **Intrauterine balloon pressure, n(%)** | 3(20) | 11(27.5) | *χ^2^=0.049, P=0.83 |

***Continuety correction;** Fisher’s exact test; AFLP,Acute Fatty Liver of Pregnancy; PIH, Pregnancy Induced Hypertension; FGR,Fetal Growth Restriction; ALT, Alanine Aminotransferase; TBA, Total Bile Acid; ICU, intensive care unit.**
