# Supplementary material for: Pregnancy outcomes of patients with acute fatty liver of pregnancy: a case control study
Source: BMC Pregnancy Childbirth. 2020 May 11;20:282. doi: 10.1186/s12884-020-02980-2 (PMC7216501; doi:10.1186/s12884-020-02980-2)
Supplement: Supplementary file 3 — Additional file 3 Suppl Table 3. Maternal outcomes in patients received intrauterine balloon pressure or not when postpartum hemorrhage exceeding 500 ml(n = 28) [file 12884_2020_2980_MOESM3_ESM.doc]

**Supp table 3. Predictors of Negative Fetal and Infant Outcomes in mothers with AFLP(n=61)**

| **Maternal Baseline Characteristics** | **Fetus with Negative outcomes (n=17)** | **Fetus without Negative outcomes (n=44)** | **t/Z/χ2, P** |
| --- | --- | --- | --- |
| **Age (mean±SD, years)** | 27.00±2.57 | 29.21±5.28 | t=2.1, P=0.042 |
| **Gravidity , n(%)** | | | |
| 1 | 9(52.9) | 23(52.3) | Z=0.12, P=0.90 |
| 2 | 5(29.4) | 12(27.3) |
| >2 | 3(17.6) | 9(20.5) |
| **Multiparae, n(%)** | 7(41.2) | 19(43.2) | χ2=0.02, P=0.89 |
| **Gestational weeks of diagnosis(mean±SD)** | 36.21±3.27 | 35.94±2.23 | t=0.35, P=0.73 |
| **Complications before the AFLP onset, n(%)** | | | |
| PIH | 4(23.5) | 9(20.5) | χ2=0.069, P=0.79 |
| singleton | 17(100) | 32(72.7) | *χ2=4.2, P=0.041 |
| FGR | 1(5.9) | 3(6.8) | χ2=0.018, P=0.90 |
| **Lab on first visit(mean±SD)** | | | |
| Platelet (*10^9^/L) | 141.35±63.90 | 122.89±70.81 | t=0.94, P=0.35 |
| Hemoglobin(g/L) | 107.29±25.70 | 103.43±21.27 | t=0.60, P=0.55 |
| ALT(IU/L) | 328.80±277.48 | 164.60±194.25 | t=2.6, P=0.011 |
| TBA(umol/L) | 109.40±73.96 | 77.45±45.39 | t=1.8, P=0.083 |
| Albumin(g/L) | 26.25±3.44 | 24.92±5.19 | t=0.16, P=0.25 |
| Total Bilirubin(umol/L) | 208.46±108.89 | 130.63±107.46 | t=2.5, P=0.014 |
| Prothrombin activity(%) | 29.51±23.10 | 46.50±22.31 | t=2.6, P=0.011 |
| C[reatinine](http://www.youdao.com/w/creatinine/#keyfrom=E2Ctranslation)(umol/L) | 166.84±81.76 | 156.86±90.73 | t=0.40, P=0.69 |
| Hypoglycemia (%) | 10(58.8) | 27(56.8) | χ2=0.02, P=0.88 |
| **Placenta abruption, n(%)** | 3(17.6) | 4(9.1) | *χ2=0.24, P=0.62 |
| **C[esarean s](http://dict.youdao.com/w/cesarean delivery/" \l "keyfrom=E2Ctranslation)ection, n(%)** | 13(76.5) | 38(86.4) | χ2=0.88, P=0.35 |
| **Preventive plasma transfusion, n(%)** | 12(70.6) | 12(27.3) | χ2=9.6, P=0.002 |
| **Intrauterine balloon pressure, n(%)** | 8(47.1) | 7(15.9) | χ2=6.4, P=0.011 |
| **Negative maternal outcome, n(%)** | 7(41.2) | 8(18.2) | χ2=3.5, P=0.061 |

***Continuety correction; AFLP, Acute Fatty Liver of Pregnancy; PIH, Pregnancy Induced Hypertension; FGR, Fetal Growth Restriction; ALT, Alanine Aminotransferase; TBA, Total Bile Acid.**
